# Case Report: Gastric Carcinoma Diagnosed at the Second Trimester of Pregnancy

**DOI:** 10.1155/2011/532854

**Published:** 2011-07-14

**Authors:** Tayfur Cift, Begüm Aydogan, Murat Akbaş, Burcu Aydın, Fuat Demirkiran, Dogu V. Bakkaloglu, Sennur Ilvan

**Affiliations:** ^1^Department of Gynecology and Obstetrics, Cerrahpaşa Medical School, Istanbul University, 34303 Istanbul, Turkey; ^2^Department of Pathology, Cerrahpaşa Medical School, Istanbul University, 34303 Istanbul, Turkey

## Abstract

We report a rare case of gastric cancer in pregnancy. A 26-year-old woman presented at the 20th week of pregnancy complaining of nausea and vomiting. Although the patient considered the condition to be related with pregnancy and underestimated its importance, her complaints persisted over the following weeks and she was hospitalized for investigation. The diagnostic workup revealed a metastatic gastric cancer. Gastric cancer is very rare in pregnancy, and therefore it may be left out of differential diagnosis by physicians. Diagnosis may be further delayed because of overlapping symptoms occurring during normal pregnancy (nausea, vomiting, and fatigue). All these factors may contribute to a very high mortality of this malignancy during pregnancy.

## 1. Introduction

Gastric cancer with pregnancy is quite rare and often diagnosed at an advanced stage [1]. Moreover, there is a controversy about management since treating an aggressive maternal malignancy during an ongoing pregnancy is a two-edged sword. In many cases, it is diagnosed at an advanced stage, making a very difficult to ensure a cure for the mother while continuing with the pregnancy. The treatment plan of gastric cancer with viable pregnancy should be promptly formulated in accordance with the number of weeks of gestation. 

We report a case of advanced gastric cancer that was diagnosed during 24th week of gestation. The disease was accepted inoperable and systemic 5-Fluorouracil + Ca leucovorin chemotherapy was given at 29 weeks of gestation. The fetal ultrasonography and fetal doppler studies did not reveal any pathology.

Although gastric carcinoma is rare under the age of 30, it must be included in the differential diagnosis of epigastric discomfort especially in the presence of persistent nausea, vomiting, and weight loss beyond the first trimester in pregnancy. Early detection of gastric cancer in pregnancy still remains to be elusive because the initial symptoms are usually nonspecific and they may easily be attributed to normal course of pregnancy. Endoscopic evaluation is recommended for complicated cases such as atypical and severe refractory dyspepsia continuing beyond the 16th week of gestation, and nausea and vomiting that do not respond to traditional treatment modalities. 

We present a case of advanced stage gastric carcinoma which was diagnosed at the second trimester of pregnancy.

## 2. Case

**Figure 1:**
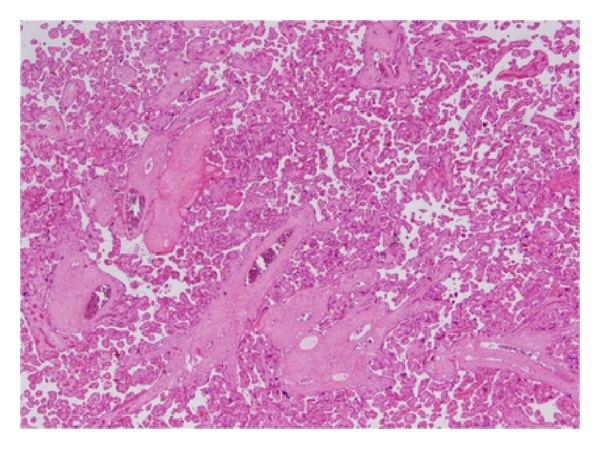
Findings compatible with the third trimester: small villi with prominent syncytiotrophoblasts, areas with thinned syncytiotrophoblast adjacent to blood vessels (vasculosyncytial membranes), and much less apparent cytotrophoblasts. (HE ×40).

A 26-year-old, gravida 2 para 1 pregnant woman admited to our clinic with intractable abdominal pain, nausea and vomiting at 20th week of pregnancy. She had recurrent attacks of nausea and vomiting before 20th week. During followup, nausea and vomiting severity was increased and became less responsive to medical treatment. Because of abdominal distention, ascites cytological evaluation of ascite fluid was performed. Cytology reported signed ring cell carcinoma. Gastroduedonoscopy was performed and linitis plastica was seen. Biopsy was reported, as previous one, signed ring cell carcinoma. Bilateral adnexial masses, pleural effusion and ascites were visualized in abdominal MRI scan. Tumour markers were as follows; CA125: 149, CA19-9: <1200, and CA15-3: 25. She was decided to be given a five day treatment of 425 mg/m^2^ 5-FU and 10 mg/m^2^ calcium folinat until delivery. On the fourth day of chemotherapy, baby was born by vaginal delivery at 29th gestational age. Its birth weight was 930 grams. The APGAR score was 3 and 5 at first and fifth minutes, respectively. Clinical condition of patient became worse after treatment and could survive two days after end of chemoterapy. The pathological examination of placenta showed no metastasis (Figure 1).

## 3. Discussion

Gastric cancer in pregnancy is rare and often not diagnosed until the very late stages [1]. The prognosis is usually grave. One of the reasons is that the mild gastrointestinal symptoms are common during pregnancy, and the other is initial symptoms are nonspecific to cancer [2]. 

Pregnancy associated gastric cancer is currently attributed to 0.1% of all cases [1]. There is an increase risk of diffuse and scirrhous type carcinomas in this group of patients. The initial diagnosis of gastric carcinoma is often delayed because up to 80% of patients are asymptopatic during early stages of gastric cancer [3]. 

The prognosis of gastric cancer during pregnancy is poor [4]. One reason for this is most gastric cancers occurring during pregnancy are advanced in stage; the diagnosis is delayed because symptoms are attributed to pregnancy and, moreover, they are all nonspecific for cancer [1]. 

Another reason is that patients of child-bearing age seem to have a greater potential for aggressive gastric cancer, which is poorly differentiated adenocarcinoma with scirrhous type of growth and peritoneal metastasis just as in our case. The third reason is the difficulty of saving both mother and fetus. The situation has two aspects. One of them is the management of pregnancy, chemotherapy usage, and surgery during the ongoing pregnancy. The other side is that the prognosis of the cancer and survival of the mother. 

Although, gastric cancer is more frequently seen in elderly population with a mean age of 60 years and predominates in males with a male : female ratio of 1.7 : 1. On the other hand, among patients under 40 years of age, it is found to be more common in females and more aggressive with a male : female ratio of 1 : 1.5 [5]. As a conclusion, young gastric cancer cases are characterized by preponderance to female sex, proximal localization of the tumor, poor differentiation, more agressive clinic, and overall poorer prognosis [6]. 

The pathogenesis of pregnancy-related gastric cancer is an issue of debate. There are conflicting data about the hypothesis. On the other hand, pregnancy itself is suggested an etiologic factor due to hormonal status. Furukawa et al. demonstrated experimentally a suppressive effect of sex hormones on spreading of stomach cancer in rat models. Immunosuppressive influence of pregnancy may be an additional factor in the development of the malignant process. Moreover, he published a data of 64 female patients and concluded that pregnancy and/or delivery in young females accelerates the growth of stomach cancer [2]. On the other hand, Jaspers et al. suggested that clinical features and prognosis in pregnancy gastric cancer cases were not significantly different from other young patients [7]. 

Lanciers et al. reported as the *H. pylori* infection rate is significantly higher for pregnant women (26%) than nonpregnant women (11%). Furthermore, in pregnancy the acid secretion in stomach decreases and mucus production increases [8]. Additionally, placenta secretes histaminase which degrades histamine function, hence the patient shows no deterioration of symptoms caused by the cancerous ulcer. As it is known, blood circulation increases during pregnancy, pregnant women are particularly susceptible to the rapid growth and spread of cancer. 

Bačići et al. reported a rare presentation of acute cardiac tamponade in a patient with gastric cancer in pregnancy. In the case, symptoms were interpreted initially by the patient as a condition related to the normal state of pregnancy. They point out gastric cancer is very rare in pregnancy, and therefore may be not suspected by physicians. Diagnosis may be further delayed because of overlapping symptoms occurring during normal pregnancy (nausea, vomiting, and shortness of breath) [9]. 

Even though, the pregnancy associated gastric cancer is rare and there have been only three reported cases with metastasis to placenta or the fetus. Placental or fetal metastasis from maternal origin of malignancy is rare [5]. In our case, according to pathologic evaluation, there was no placental metastasis. 

Early detection of gastric cancer in pregnancy still remains to be a dilemma due to infrequency of the disease in young population and misinterpretation of the nonspecific symptoms. Endoscopic examination has been reported to be safe in pregnancy and recommended for complicated cases such as atypical and severe refractory gastrointestinal symptoms continuing beyond the 16th week of gestation, nausea-vomiting that does not respond to standard treatment modalities, or suspicious symptoms of malignancy [10]. Additionally, the risk factor group for gastric malignancy also must be taken into account in pregnant women. 

Management during pregnancy can be lined according to the stage of the tumor and the gestational age of the fetus. In inoperable cases, chemotherapy is the first choice.

Since it has been known that the prognosis in gastric cancer is poor, 80% die in the first year and 3-year survival rate is 8%, early diagnosis is critical. Early recognition and diagnosis is the only possibility for a better outcome. 

Although it is rare under the age of 30, it must be included in the differential diagnosis of epigastric discomfort, nausea, vomiting, and weight loss beyond the first trimester of pregnancy. 

Endoscopic examination should not be delayed in suspected cases. If disease is operable, suitable time for operation must be decided according to the gestational age of fetus. Above all, if it is inoperable, chemotherapy is the first choice and a proper regimen must be given considering the fetus.
